# A Report of the Surgeons’ Congress

**Published:** 1914-09

**Authors:** Joe Gilbert

**Affiliations:** Austin, Texas


### A Report of the Surgeons' Congress.

BY DR. JOE GILBERT, AUSTIN, TEXAS.

The session of the Clinical Congress of Surgeons opened July 27th with about 1500 in attendance, drawn from every part of the globe. The first day all the hospitals in London were thrown open, and one was able to find any kind of surgical work that he might wish. Lists of operations were posted then and each succeeding day at headquarters at the Hotel Cecil, and cards issued to the surgeons for any clinics they chose to attend. Needless to say, they were operations of every kind on every part of the body.

The formal opening on the evening of the 27th was imposing. We were welcomed by Sir Rickman J. Godlee, Honorary of the London Committee; also an address of welcome, directed particularly to the American surgeons, from the Hon. Walter Hines Page, American Ambassador. We had a very interesting paper from Prof. A. Von Eiselberg, of Vienna, on "The Choice of the Operative Method for Ulcer of the Stomach." But, to my mind, the feature of the evening was the reception of our distinguished countryman, John B. Murphy, the President of the Congress. He took the house by storm with his address on "Arthrodesis and Bone Transplantation: Its Limitations and Technique." He had them all surpassed on expressing and elucidating his vast fund of experience.

I selected Guy's Hospital for my first day's work. Among others, I saw Sir Arbuthnot Lane perform his favorite operation on the intestine; i. e., the removal of the entire large intestine for intestinal stasis. He is very decisive in his views in regard to this, and, while the majority of surgeons do not agree with him fully (that is, in the removal of the entire colon for the cure of such a wide range of diseases), still they realize that he has located the cause of quite a number. He himself admits, however, that the method is a little radical, but is the best way discovered, he thinks, up to the present time, to remove the cause of many diseases in the advanced stages. Those diseases which he claims to cure by this method are all forms of rheumatism (including rheumatoid arthritis), gastric and duodenal ulcers, diseases of the gall bladder, tuberculosis of intestinal canal, goitre, etc.

At Guy's I saw Mr. C. H. Fagge doing some nerve anastomosis for paralysis of the facial nerve. He reported having been successful in a number of cases in restoring it to its normal function. Guy's is a very old hospital, built in 1721, but the surgeons are doing excellent work there still.

At the London Hospital, one of the oldest and largest hospitals there, with its modern addition and facilities, I saw such men as Sir Frederick Eves, Howard, Rigby (physician to the King), Milne, Walton (the last three comparatively young men), all perform difficult operations in a most scientific manner. In one morning, Mr. Walton's program was as follows:

1. Thyroidectomy.
2. Resected a gastric ulcer in the posterior cardiac region of stomach. Did a pyloroplastic and a posterior gastro-enterostomy on the same subject.
3. A gastro-enterostomy—then through a second incision, an ovariotomy.
4. Posterior gastro-enterostomy for duodenal ulcer and removal of appendix through the same incision.
5. Removal of gall bladder for gall stones.

He began at 9 a. m. and finished at 12:30; did all the work himself, even to closing of abdomen.

Sir Berkley Monyhan, of international reputation for intestinal surgery, made an address. Here let me say that I consider him the best surgeon that I saw at work. I made a special trip to his home city—Leeds—to see him before the opening of the Congress. His methods are like the American surgeons (which may have something to do with my admiration). His address was on intestinal stasis, and was practical and up to date. He disagreed with Sir Arbuthnot Lane on indiscriminate removal of colon.

One little item that was typical of England was the interruption of the meeting by a suffragette, a militant one. It was at a very interesting point in the paper of Dr. Rodman, of Philadelphia, President of the American Medical Association, on "Breast Tumors," that she appeared in the midst of the audience and began to orate in a loud manner. The American surgeons good naturedly "hurrahed" her until the arrival of a policeman.

Among the most modern hospitals I saw, besides those of London, were two at Edinburgh, of about 600 beds each, and two at Glasgow. Among them, I visited the hospital where Lister established antiseptic surgery. Sir Wm. McEwen, known chiefly through his teachings in bone surgery, at the age of 73, is still doing fine work at Glasgow University.

To sum it all up, I would say that the surgeons over there have a more thorough preliminary education than ours as a class. Of course, there are exceptions to this among our surgeons, but a sounder training is required of an English surgeon. They are great anatomists and pathologists, depending more on their clinical symptoms for diagnosis. In work, they are very dexterous, using their hands a great deal, particularly in needle work, using needle-holders very little. Most of them use silk for intestinal anastomosis, using silk more generally than the American surgeons.

